# Characteristics of Antivaccine Messages on Social Media: Systematic Review

**DOI:** 10.2196/24564

**Published:** 2021-06-04

**Authors:** Dominik Wawrzuta, Mariusz Jaworski, Joanna Gotlib, Mariusz Panczyk

**Affiliations:** 1 Department of Education and Research in Health Sciences Faculty of Health Sciences Medical University of Warsaw Warsaw Poland

**Keywords:** vaccination, social media, antivaccination movement, vaccination refusal, health communication, public health, vaccines

## Abstract

**Background:**

Supporters of the antivaccination movement can easily spread information that is not scientifically proven on social media. Therefore, learning more about their posts and activities is instrumental in effectively reacting and responding to the false information they publish, which is aimed at discouraging people from taking vaccines.

**Objective:**

This study aims to gather, assess, and synthesize evidence related to the current state of knowledge about antivaccine social media users’ web-based activities.

**Methods:**

We systematically reviewed English-language papers from 3 databases (Scopus, Web of Science, and PubMed). A data extraction form was established, which included authors, year of publication, specific objectives, study design, comparison, and outcomes of significance. We performed an aggregative narrative synthesis of the included studies.

**Results:**

The search strategy retrieved 731 records in total. After screening for duplicates and eligibility, 18 articles were included in the qualitative synthesis. Although most of the authors analyzed text messages, some of them studied images or videos. In addition, although most of the studies examined vaccines in general, 5 focused specifically on human papillomavirus vaccines, 2 on measles vaccines, and 1 on influenza vaccines. The synthesized studies dealt with the popularity of provaccination and antivaccination content, the style and manner in which messages about vaccines were formulated for the users, a range of topics concerning vaccines (harmful action, limited freedom of choice, and conspiracy theories), and the role and activity of bots in the dissemination of these messages in social media.

**Conclusions:**

Proponents of the antivaccine movement use a limited number of arguments in their messages; therefore, it is possible to prepare publications clarifying doubts and debunking the most common lies. Public health authorities should continuously monitor social media to quickly find new antivaccine arguments and then create information campaigns for both health professionals and other users.

## Introduction

### Background

From the time of its inception, the internet has provided supporters of the antivaccine movement with unprecedented possibilities to affect societies by nearly global communication of their opinions and convictions. The internet has immense potential as a medium for disseminating health information. However, this information can be unreferenced, incomplete, or informal, and thus, it can be considered dangerous [1-5]. The antivaccination messages on the internet are far more unbridled than in other media. For this reason, the internet is a source that can lead to the risk of people making uninformed decisions about vaccination [6]. Contemporary social media is regarded as a major communication tool for spreading information about the antivaccination movement [7,8].

Early studies concerning the content shared on antivaccination websites revealed that this thematic area includes 3 main issues: concerns about vaccine safety and effectiveness, concerns about governmental abuses, and a preference for alternative health practices [2,6,9,10]. First, a key aspect is the perceived risk of severe and long-term side effects of vaccinating a child. Second, mandatory vaccinations are perceived as an unacceptable breach of the right to make independent decisions and as a limitation of civil liberties. A mistrust also exists that is expressed through the belief that governmental supervision bodies keep the reports about the adverse reactions associated with vaccinations a secret and collaborate with the pharmaceutical industry to profit from vaccine sales. Third, alternative health practices are promoted as being more natural than conventional medicine, and they are believed to eliminate the need for vaccination once they are properly followed.

The majority of the antivaccine arguments evoke negative emotions such as fear [8]. In contrast, many provaccine arguments and campaigns are grounded in the values of harm and fairness [11]. Furthermore, the moral ideas of purity and liberty are mostly associated with vaccine hesitancy. The people for whom these values are of great significance will be insensitive to the information on the positive aspects of vaccinations [12]. Most of the arguments used by antivaccination activists can be perceived as part of a broader phenomenon called *denialism*. Denialism can be defined as “the employment of rhetorical arguments to give the appearance of a legitimate debate where there is none, an approach that has the ultimate goal of rejecting a proposition on which a scientific consensus exists” [13]. The European Center for Disease Prevention and Control defines vaccine hesitancy as a “delay in acceptance or refusal of vaccines despite availability of vaccination services” [14]. Such a delay results from broader influences and should always be considered in the historical, political, and sociocultural contexts in which vaccinations occur [15-17]. According to the World Health Organization, vaccine hesitancy is a continuum with the people who are unsure about vaccines or want to delay only some vaccinations being on one end and people who refuse all vaccines being on the other [18].

The spread of negative information about vaccination on the internet and social media is considered to be the leading cause of vaccine hesitancy [17]. Many studies have revealed the negative impact of media controversies related to vaccination safety on the level of vaccination coverage [19,20]. The ubiquitous presence of antivaccination content on the World Wide Web contributes to the prompt dissemination of rumors, myths, and false opinions about vaccines, which subsequently lowers vaccination coverage [4,10,21]. The results of a study by Betsch et al [22] revealed that reading antivaccination webpages for even approximately 5-10 minutes negatively affects the perception of the risk related to vaccination. Dube et al [23] and Smith et al [24] made similar observations. Glanz et al [7] emphasized that exposure to antivaccine messages through social media may intensify parents’ worries and change their intention to vaccinate their children.

Web 2.0 functions (such as those in Facebook, Twitter, YouTube, and blogs and discussion groups) enable users to develop and share content; thus, users can easily and quickly share their personal experiences related to vaccination [21,25]. In this context, social media has become a platform where intimate and emotionally loaded information is exchanged [26]. Here, one can also find the support of people with similar health problems or doubts [27]. Some studies have revealed that content related to vaccines can be found in all social media and that the critical content generally correlated with the content previously published on an antivaccination websites [28,29]. Several important claims on social media may have promoted this phenomenon, including claims that social media is a *big business* and is powerful, that the audience is poorly defined, that fairness is irrelevant, and that nothing is truly private [30]. These factors may affect people’s decisions concerning treatment and preventive health care, including preventive vaccination.

Searching for health information on social media has become increasingly popular [26,31]. Analyses revealed that 41% of parents found antivaccination content through social media [32]. Nearly 16% of all internet users look for information about vaccinations, and 70% of them confirmed that the information they had found affected their decisions [33]. Evrony and Caplan [8] emphasized the need for a more effective search and criticism of the false information on vaccination published on the internet. They highlighted that although every internet user has a choice, spreading disinformation and distorting the facts concerning vaccines is not a choice. Such activities should be noted and effectively challenged. Therefore, it is crucial to learn not only about antivaccine information but also about its emotional load and to identify the content addressees. Such a thorough analysis will help to better understand the motivations and emotions that accompany the information created by antivaccinationists.

Currently, the literature is limited to review articles on the potential role of social media in influencing vaccination beliefs and behavior [10,21,33]. Despite the growing number of papers studying the content published by supporters of the antivaccination movement in social media in recent years, no systematic review analyzing these papers has been developed so far. Previous literature reviews have analyzed disinformation concerning health on social media [34] and health information shared on YouTube [35]. They tackled the issue of vaccination and antivaccination movements only on a fragmented basis, as part of a broader topic. Catalan-Matamoros and Peñafiel-Saiz [36] published a systematic review investigating the communication about vaccinations in traditional media, excluding the content shared on the internet and social media. Considering the activity of the antivaccination movement’s supporters on social media and how easily they can communicate their messages that are not scientifically confirmed to a large number of recipients, it is crucial to learn and understand their activities and messages. This knowledge will help us react and respond effectively to the false information they publish.

### Objectives

This study aims to gather, assess, and synthesize evidence regarding the current state of knowledge about antivaccine social media users’ web-based activities. We checked 4 general hypotheses about the features of antivaccine content on social media that are often described in papers in the field of vaccinology:

1. Antivaccine messages are more liked and shared than provaccine content.
2. Antivaccine content is more user friendly than provaccine content.
3. Antivaccine social media users describe vaccines as harmful for health or ineffective.
4. Antivaccine social media users share conspiracy theories or claims that are not scientifically proven.

In addition, we summarized the current knowledge on the role of social media bots in the spread of antivaccine messages.

## Methods

### Design

We designed a systematic review that systematically searched, appraised, and synthesized research evidence following the PRISMA (Preferred Reporting Items for Systematic Reviews and Meta-Analyses) guidelines [37].

### Eligibility Criteria

We included papers based on the following inclusion criteria:

1. Papers based only on original studies.
2. Papers analyzing the user-generated content available on social media platforms (including Twitter, Facebook, Instagram, Pinterest, YouTube, LinkedIn, TikTok, Snapchat, VKontakte, WhatsApp, WeChat, Tumblr, Qzone, Reddit, and Sina Weibo).
3. Papers in which the authors extracted a group of antivaccine messages and conducted analyses that contained both qualitative and quantitative elements.

The exclusion criteria were as follows:

1. Papers not written in English.
2. Papers in which vaccines were not the main topic of research.
3. Methodological papers.
4. Conference abstracts.

### Information Sources and Search Strategies

Our search strategy was guided by the following research question: *What are the existing data about antivaccine users’ activity on social media*? We conducted a scoping literature search of papers published between January 1, 2015, and December 31, 2019, on 3 databases (Scopus, Web of Science, and PubMed), taking into account the PRISMA guidelines. Overall, 2 reviewers (DW and MP) independently screened the databases using the same protocol. The search was conducted in January 2020. We chose the last 5 years for two reasons. First, in 2014, the social media market began to resemble the one known today. The growth of the major platform Facebook slowed, but the activity of users increased. Furthermore, smaller platforms such as Twitter, Instagram, Pinterest, and LinkedIn gained popularity [38]. Second, from our abstract analysis, we found no articles published in 2014 that were potentially eligible for this systematic review; this suggests that the number of papers analyzing negative messages on social media started to grow in 2015.

The query used in the search engines of the databases is as follows:

( ( “social media” OR “Twitter” OR “Facebook” OR “Instagram” OR “Pinterest” OR “YouTube” OR “LinkedIn” OR “TikTok” OR “Snapchat” OR “VKontakte” OR

“WhatsApp” OR “WeChat” OR “Tumblr” OR “Qzone” OR “Reddit” OR “Sina Weibo” )

AND

( “vaccin*” OR “immuni*” ) )

We also searched the reference lists of the previously retrieved studies and literature reviews.

### Study Selection

**Figure 1:**
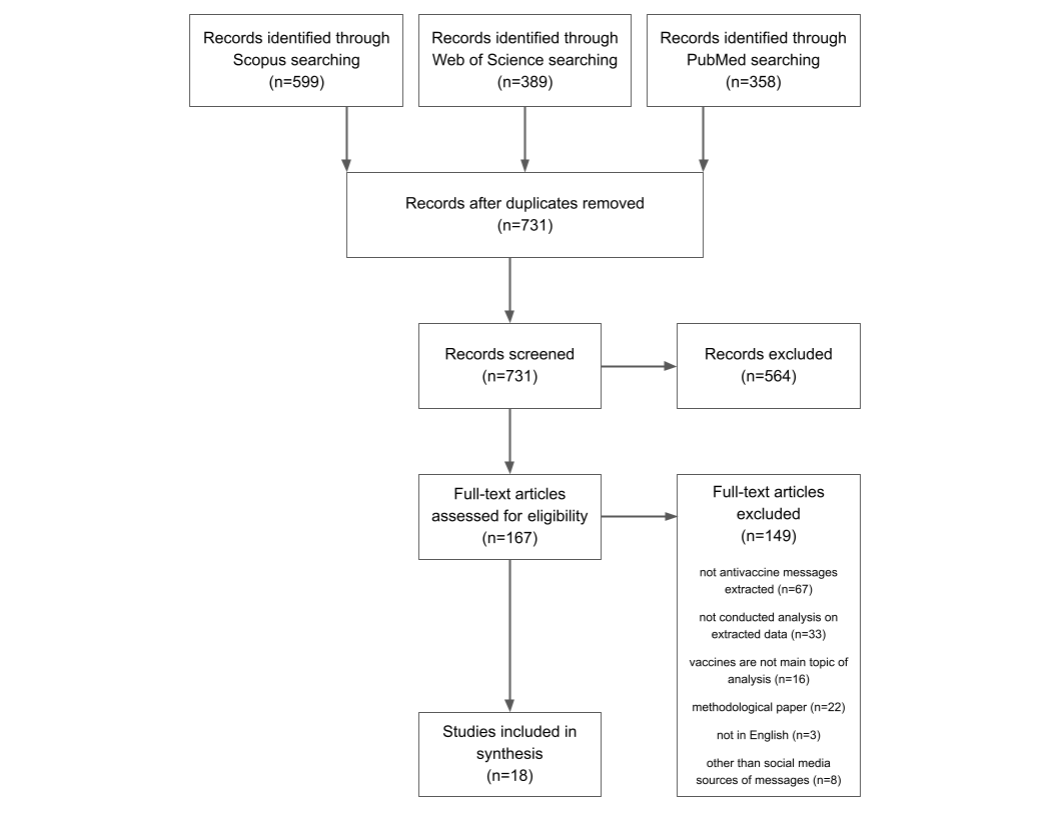
Preferred Reporting Items for Systematic Reviews and Meta-Analyses flow diagram.

A 3-stage approach was used to include and exclude studies in the final review process. Initially, duplicate studies were excluded; subsequently, a screening was performed based on the (1) title, (2) abstract, and finally, (3) full text. Discrepancies regarding article selection were resolved by a consensus within the reviewing team (Figure 1).

### Data Collection Process, Extraction, and Quality Assessment

We found 599 articles from Scopus, 389 from Web of Science, and 358 from PubMed. After removing duplicates, 731 articles were analyzed further. In the next step, the titles, abstracts, and keywords were screened. At this stage, we removed papers dealing with pharmacovigilance on social media, survey research, theoretical mathematical models, interventions in social media to increase vaccination coverage, and descriptive analysis without the messages being extracted. Two researchers (DW and MP) conducted a full-text analysis of the remaining 167 papers. A data extraction form was established, and it included authors, year of publication, country, specific objectives, study design, social media platform, type of data analyzed, number of messages studied, main objectives and findings, and geographic focus. Standardized checklist tools were not used to assess the quality and risk of bias of individual studies.

### Synthesis of the Results

The outcomes of the 18 studies included in the final analysis were heterogeneous. We performed an aggregative narrative synthesis of the included studies.

## Results

### Studies’ Characteristics

The number of articles analyzing antivaccination messages on social media has increased over the last 5 years. This topic became highly popular in 2019, during which 7 of the 18 included papers were published. Overall, 7 of the 18 papers studied Twitter. YouTube was slightly less popular and was analyzed 6 times. The remaining articles studied Facebook, Instagram, and Pinterest. Although most of the authors analyzed text messages, some of them studied images or videos. The number of analyzed messages varied from 123 to 1,793,690 across the papers. The oldest messages were published in 2006. Table 1 describes the general characteristics of the included studies.

**Table 1:** Characteristics of the included studies.

Study	Platform	Type of data	Number of messages	Geographic focus	Main topic	Time interval
Basch and MacLean (2019) [39]	Instagram	Text, image	150	English language	HPV^a^	2018
Blankenship et al (2018) [40]	Twitter	Text	1626	English language	Not specified	2014-2015
Broniatowski et al (2018) [41]	Twitter	Text	1,793,690	English language	Not specified	2014-2017
Covolo et al (2017) [42]	YouTube	Video	123	Italian language	Not specified	2014-2015
Donzelli et al (2018) [43]	YouTube	Video	560	Italian language	Not specified	2007-2017
Ekram et al (2019) [44]	YouTube	Text, video	35 videos with comments	English language	HPV	2014
Faasse et al (2016) [45]	Facebook	Text	1489	English language	Not specified	2016
Guidry et al (2015) [46]	Pinterest	Images	800	English language	Not specified	2014
Harvey et al (2019) [47]	Facebook	Meme	234	English language	Not specified	Not specified
Kang et al (2017) [48]	Twitter	Text	26,389	United States	Not specified	2015
Kearney et al (2019) [49]	Instagram	Text, image, video	360	English language	HPV	2011-2018
Luo et al (2019) [50]	Twitter	Text	287,100	English language	HPV	2008-2017
Massey et al (2016) [51]	Twitter	Text	193,379	English language	HPV	2014-2015
Mitra et al (2016) [52]	Twitter	Text	315,240	English language	Not specified	2012-2015
Song and Gruzd (2017) [53]	YouTube	Video	1984	English language	Not specified	Not specified
Venkatraman et al (2015) [54]	YouTube	Video	175	English language	Not specified	Not specified
Yiannakoulias et al (2019) [55]	YouTube	Video	275	English language	Influenza, measles	2006-2018
Yuan et al (2019) [56]	Twitter	Text	669,136	English language	Measles	2015

^a^HPV: human papillomavirus.

There are differences in the methods used to extract the messages. Most of the authors searched for general vaccine-related queries [41,42,46-48,52] or the content of tags [40]. Papers analyzing the human papillomavirus (HPV) vaccine used both general terms (such as *HPV*, *HPV vaccine*, *#HPV*, and *#HPVvaccine*) [39,44,49-51] and specific vaccine names (*Gardasil* and *Cervarix*) [49,51]. Polarized opinions, both positive and negative, were isolated using phrases containing words connected to vaccination and autism [43,53,54]. As Facebook does not allow for searching of posts using a search engine, the authors analyzing this platform used more sophisticated methods to extract messages. Faasse et al [45] studied responses to a photograph that promoted vaccination and was published by Facebook CEO Mark Zuckerberg. Harvey et al [47] found 128 Facebook fan pages devoted to vaccination and sampled up to 30 memes from each page. The papers included content analyzed at different time intervals (Table 1). Only 1 study used search criteria specifically related to the outbreak of an infectious disease. Yuan et al [56] used Twitter data from February 1 to March 9, 2015, to study the tweets published just after the measles outbreak in Disneyland, California, in 2015.

Although most studies examined vaccines in general, 5 focused specifically on HPV vaccines, 2 on measles, and 1 on influenza. Among the articles examining negative comments about the HPV vaccine, 2 examined Twitter, 2 Instagram, and 1 YouTube. These HPV-related messages mention the side effects and risks associated with vaccination [50,51] but often omit some information [44]. Furthermore, HPV-related antivaccine messages on Instagram used more sophisticated forms of communication, including videos or text with images [49]. Provaccine content describing the HPV vaccine contained information on protection against and prevention of cancer [39,50,51] and the safety of the vaccine [44].

In Table 2, we have aggregated the research questions of the included studies into 4 general hypotheses. In the next paragraphs***,*** we describe the results of the analyzed articles in terms of these hypotheses and we discuss bots’ activities on social media.

**Table 2:** Results of the included studies.

Study	Hypothesis 1: Antivaccine messages are more liked and shared than provaccine content	Hypothesis 2: Antivaccine users share more user-friendly content than provaccine users	Hypothesis 3: Antivaccine users describe vaccines as harmful for health	Hypothesis 4: Antivaccine users share conspiracy theories or claims that are not scientifically proven
Basch and MacLean (2019) [39]	Antivaccine messages have more likes than provaccine content (*P*=.02)	N/A^a^	Antivaccine users are less likely to mention the protection provided by the HPV^b^ vaccine (*P*<.001)	N/A
Blankenship et al (2018) [40]	Antivaccine messages have more retweets than provaccine ones (*P*<.001)	Most shared antivaccine-related URLs link to Twitter, YouTube, and Facebook	N/A	N/A
Broniatowski et al (2018) [41]	N/A	Content polluters, which may have been primarily created for marketing, spread more antivaccine content than the average nonbot users (*P*<.001)	N/A	N/A
Covolo et al (2017) [42]	Antivaccine messages are more liked (*P*<.001) and shared (*P*<.001) but are less viewed (*P*<.001) than provaccine content	N/A	Fear-related themes are present more often in the antivaccine content than in the provaccine one. However, the difference is not statistically significant (*P*>.05)	Antivaccine content is less likely to have been developed by health professionals than provaccine contents (*P*=.002)
Donzelli et al (2018) [43]	Antivaccine messages have more likes, shares, and views (*P*<.001) than provaccine content	Antivaccine content is mainly represented in the categories “People & Blogs” and “No-profit and activism”	N/A	N/A
Ekram et al (2019) [44]	There is no difference between anti- and provaccine content in the number of likes and views (*P*>.05)	N/A	Antivaccine users are more likely to exclude information about vaccine safety (*P*<.05) and efficacy (*P*<.05)	Antivaccine users are more likely to report incorrectly (*P*<.001) or omit information (*P*<.01)
Faasse et al (2016) [45]	Antivaccine comments have fewer likes than provaccine comments (*P*<.001)	Antivaccine content uses more analytical thinking (*P*<.001) and less tentative language (*P*=.055)	Antivaccine users use more words related to health (*P*<.001) and money (*P*=.03)	N/A
Guidry et al (2015) [46]	N/A	Antivaccine users use more narrative than statistical information (*P*<.001)	N/A	27.7% of the antivaccine content mentions conspiracy theories
Harvey et al (2019) [47]	Antivaccine messages get more reactions (*P*<.001) and shares (*P*<.001) than provaccine content	Antivaccine content has stronger emotional appeal (*P*<.001)	Antivaccine content appeals more to fear (*P*<.001) and more often uses themes connecting vaccines with injuries, safety issues, and autism (*P*<.001)	Antivaccine content contains a greater percentage of false statements (*P*<.001) and more often mentions conspiracy theories (*P*<.01)
Kang et al (2017) [48]	N/A	Antivaccine content addresses a broader range of topics compared with provaccine content	Central concepts are thimerosal, mercury, autism, flu shots, and vaccine ingredients	Central concepts are CDC^c^, the vaccine industry, mainstream media, doctors, mandatory vaccines, and pharmaceutical companies
Kearney et al (2019) [49]	Antivaccine messages are more liked (*P*<.001) than provaccine ones	Antivaccine content uses more personal narratives than informational ones (*P*<.001). Videos or text with images are more often used rather than text or images alone (*P*<.001)	N/A	Antivaccine content is less likely to have been created by health-related users (*P*<.001)
Luo et al (2019) [50]	N/A	N/A	Antivaccine users more often use words such as “death,” “concern,” “kill,” “injured,” “safety,” “adverse,” “scandal,” and “fraud”	N/A
Massey et al (2016) [51]	Negative messages have fewer retweets than provaccine ones do but are still more than neutral (*P*<.001)	N/A	Antivaccine users more often mention side effects (*P*<.001) and are less likely to discuss the protection provided by HPV vaccine (*P*<.001)	N/A
Mitra et al (2016) [52]	N/A	More direct (*P*<.05) and certain language (*P*<.05) is used in antivaccine content	Antivaccine users more often discuss death concerns (*P*<.001)	The “evil government” topic is often discussed (*P*<.001)
Song and Gruzd (2017) [53]	Antivaccine messages have a higher like-to-dislike ratio (*P*=.001)	Antivaccine content is easier to access through YouTube recommendations (centrality measures *P*<0.01). It is more likely to be found in the categories “People & Blogs” and “News & Politics” (*P*<.005)	N/A	N/A
Venkatraman et al (2015) [54]	No difference was noted in terms of likes (*P*=.86) and views (*P*=.38) between anti- and provaccine content	Antivaccine users more often use celebrities in videos (*P*=.07)	Antivaccine users are more likely to use personal stories to indicate the negative effects caused by vaccinations (*P*<.001)	The links provided by antivaccine users less often lead to scientific articles (*P*=.01)
Yiannakoulias et al (2019) [55]	Antivaccine content is more liked than provaccine content is	N/A	Antivaccine content has higher frequency of the words “mercury,” “syringe,” “chemical,” and “toxic”	N/A
Yuan et al (2019) [56]	N/A	Antivaccine users prefer to communicate with users of the same opinion group	N/A	N/A

^a^N/A: not applicable.

^b^HPV: human papillomavirus.

^c^CDC: US Centers for Disease Control and Prevention.

### Hypothesis 1: Popularity of Content

The authors used different measures to estimate the popularity of antivaccine content. These measures depended on the characteristics of the platforms. Each platform allows researchers to assess user engagement in response to the content. They measured retweets on Twitter [40,51]; likes and shares on Facebook [45,47]; likes on Instagram [39,49]; and likes, dislikes, or shares on YouTube [42-44,53-55]. In addition, YouTube allows for the measurement of audience size by providing information about the number of views, which was also analyzed in a few papers [42-44,54]. In our data set, 12 studies evaluated whether antivaccine content is more popular on social media than provaccine content. Overall, 8 of them found that antivaccine content was more popular, 2 did not find a difference in popularity, and 2 found that provaccine messages were more popular. The popularity of user-generated content depends on the type of platform and can be expressed by likes, reactions, shares, retweets, or views. On Instagram, antivaccine messages had more likes than provaccine messages [39,49]. YouTube videos with negative vaccine sentiments were more appreciated by users (they had more likes, shares, or views) [42,43,53,55]. However, 2 studies did not find statistically significant differences in the number of likes and views between provaccine and antivaccine YouTube videos [44,54]. The analysis of Twitter content did not show a clear link between sentiment and popularity. Blankenship et al [40] found that antivaccine messages had more retweets than provaccine messages, whereas Massey et al [51] found the opposite. The results of the study on Facebook content were similarly ambiguous. Harvey et al [47] found that antivaccine memes had more likes and shares, whereas Faasse et al [45] showed that provaccine Facebook comments had more likes. The results suggest that although antivaccine messages are generally more popular on social media, platform-dependent differences may exist.

### Hypothesis 2: User-friendly Content

Antivaccine users present content in a way that tends to be user friendly: it grabs the users’ attention and encourages them to read the posts. Various methods are used to ensure this. The messages contain personal narratives or stories [46,49,54] and are connected with strong emotions and fear [42,47,52]. The language used is also different. Antivaccine articles use phrases such as *mandated vaccines* and *adverse effects*, whereas provaccine articles use *required vaccines* and *side effects* [48]. The language of antivaccine content is direct and certain [52] not tentative [45]. This content is rarely created by health professionals [42,49] but is well suited to the web-based environment as it uses videos or text with images rather than text or images alone [49]. Antivaccine videos also feature celebrities more often than provaccine videos do [54]. On YouTube, negative videos are mainly presented in lifestyle categories such as *People & Blogs* [43,53]. All results show that antivaccine users create their messages in a way that effectively grabs the attention of their audience.

### Hypothesis 3: Vaccines Are Unhealthy or Do Not Work

Antivaccine users often describe vaccinations as harmful to health. They connect them with serious but scientifically unconfirmed injuries, safety issues, and side effects such as autism or even death [44,47,51,54]. They refer to the use of toxic ingredients in vaccines, including mercury and thimerosal [48,55].

Antivaccine messages try to create the impression that vaccines do not protect against diseases. Not only do they present false information about vaccines [47] but they also tend to omit information about the safety and protection provided by vaccines [39,44,51]. All papers indicated that antivaccine users try to create an impression that vaccines are ineffective and dangerous to health.

### Hypothesis 4: Vaccines Are a Part of a Conspiracy

In the antivaccine discourse, vaccines are often described as part of conspiracy theories [44,46,47]. Antivaccine concepts are linked to the Centers for Disease Control and Prevention, the Food and Drug Administration, and the pharmaceutical industry hiding the truth about vaccines’ side effects or a government using vaccines to obtain control over society or to perform eugenic actions [45,48,50,52]. Antivaccine users view mandatory vaccines as a threat to their civil liberties [46,47]. They believe that as vaccinations carry risks, patients’ informed consent is necessary.

### Bots’ Activity

Using automated tools to spread vaccine-related information can be an efficient and effective way of convincing social media users. A total of 77% of the bots active on Twitter are provaccine. They are hypersocial in retweeting, and they mainly retweet from their opinion group [56]. Research on the activity of bots and trolls in a vaccine debate on Twitter showed that content polluters, which may have been primarily created for marketing, share antivaccine messages at a higher rate than average nonbot users do. Russian trolls post vaccine content at a higher rate than nonbot users do, but they share an equal number of provaccine and antivaccine messages because their purpose is to promote discord in the vaccination debate [41].

## Discussion

### Principal Findings

Regardless of the social media platform, there are similarities in the characteristics of antivaccine content. Most of the authors found that vaccine-related messages with negative sentiments had a higher number of positive reactions on social media (likes, shares, and retweets). This relationship was particularly evident on YouTube and Instagram, whereas the results from the studies on Twitter and Facebook were inconclusive. The reason for this ambiguity in the case of Facebook may lie in the sampling of the analyzed messages. Harvey et al [47] studied the popularity of memes published on 128 Facebook pages devoted to vaccinations and found that antivaccine messages received more reactions than provaccine messages. Faasse et al [45] obtained the opposite results after studying the comments posted in response to the photo uploaded by Mark Zuckerberg to promote vaccines. However, the percentage of provaccine users constituting Mark Zuckerberg’s profile followers may be higher than the percentage of provaccine users in the entire population. On Twitter, Massey et al [51] found that negative messages have fewer retweets than positive ones, but this study was limited to HPV-related tweets. Future research should investigate the popularity of antivaccine content on Twitter and Facebook. The high number of likes on and shares of antivaccine content poses the danger that ordinary users will find this information more easily and consider it to be more reliable than provaccine messages.

We also found that antivaccine users create messages in a user-friendly manner. They publish emotional personal stories using direct language. Their content is not created by health professionals, and they publish more often in the categories under lifestyle than in those connected with medicine or science. Psychological studies have shown that emotional events are remembered more accurately and for a longer time than neutral events [57]. Thus, the emotional communication of antivaccine content may have contributed to the effectiveness of the antivaccine movement. Antivaccine activists often use methods that are commonly used in marketing. Emotional stories attract the attention of neutral users [58], whereas the stirring up of fear of vaccinations leads to the inaction of the audience [59].

The proponents of the antivaccine movement call vaccines dangerous for health and ineffective. The myth-busting research pointing to vaccine safety [60,61] is not stopping the spread of false information. Some people do not trust science, considering it as a tool in the hands of governments and pharmaceutical companies [62]. Many parents believe that the risks of vaccination outweigh its benefits [63]. In countries where parents do not see children dying from infectious diseases, it is easy to think that vaccines, and not the diseases they protect against, are the problem. This phenomenon is perhaps being magnified by the fact that fear-related sources automatically attract attention [64].

Antivaccine messages often contain conspiracy theories. Previous research indicates that a belief in at least one conspiracy theory is common in society [65]. Contrary to popular opinion, conspiracism is not a product of ignorance; it can be explained by the human willingness to believe in the unseen. In a study conducted by Jolley and Douglas [62], participants who were exposed to antivaccine conspiracy theories showed less intention to vaccinate than the control group. Education can help solve this problem because preexisting knowledge about vaccination may protect against the negative effects of exposure to vaccine-related conspiracy theories on the web [66]. Only a simple debunking of misinformation can strengthen its persistence in the community [67] or even increase the harmful activity of conspiracists [68]. Clarifying parental concerns and involving parents in decisions regarding their child’s vaccination can reduce beliefs in conspiracies [69].

The analyzed papers show that bots on social media spread not only antivaccine messages but also provaccine messages. The literature differentiates between benign and malicious bots [70]. Benign bots respond automatically, aggregate content, and perform other useful actions. However, malicious bots are designed with a purpose to harm. Their task is to manipulate, mislead, and exploit to influence social media discourse. Public health authorities should not only monitor social media, detect negative bots, and fight the spread of the antivaccine content, but they should also use benign bots to communicate with the public and dispel doubts about vaccinations.

The results of the included papers are generally consistent with those of previous research examining antivaccine website content [2,6,9,10]. Similar to websites, antivaccine users of social media raise and discuss concerns about the safety and effectiveness of vaccines. They describe vaccines as harmful for health, present scientifically unconfirmed claims, and hide information about the safety and effectiveness of vaccines to strengthen their messages. This behavior can be explained by a well-known cognitive phenomenon called *motivated reasoning*, which is defined as a tendency to find arguments in favor of the conclusions we believe in, rather than those supporting what we do not want to believe in [71]. Both antivaccine websites and antivaccine social media users presented two arguments concerning the government. They believe that the government is cooperating with pharmaceutical corporations to impose universal immunization programs and is allowing these companies to profit. The second argument does not suggest evil government plans but points to the restriction of freedom of choice because of mandatory vaccines. Unlike the content of antivaccine websites, negative posts on social media do not promote alternative health practices; they simply discourage vaccinations. This may be because short and eye-catching content is preferred on social media, and this does not allow for the explanation or discussion of complex issues.

We found that most included studies were based on Twitter data. This is surprising because Facebook, YouTube, and Instagram have many more active users [72]. The reason for this disproportionate attention may lie in the simplicity of gathering data from Twitter. Twitter enables the downloading of thousands of posts using its official application programming interface [73], whereas Facebook and Instagram closed their application programming interfaces in 2018, thus preventing the automatic downloading of publicly available data from these platforms to protect users’ data against inappropriate use [74]. This was in response to the Cambridge Analytica data misuse scandal [75]. Moreover, Facebook allows for the creation of closed and private groups whose content is not available to scientists. YouTube provides researchers with easy access to the content by providing automatically generated transcripts of videos, thus facilitating text analysis. Since 2019, Pinterest has been hosting vaccine-related information only from reliable sources to halt the spread of vaccine misinformation [76]; therefore, further research into the antivaccine user’s activity on this platform since 2019 is pointless.

Web-based platforms differ in terms of how easily the antivaccine content is spread through social media. Facebook and Instagram have taken steps to stop the spread of vaccine misinformation by making it less prominent in search results; however, such misinformation has not been completely removed from these platforms [77]. Recent research shows that since 2016, interactions with content containing misinformation have reduced on Facebook but have continued to increase on Twitter [78]. This suggests that misinformation on Twitter can become a bigger problem than on Facebook. During the COVID-19 pandemic in 2020, Twitter started removing accounts spreading health conspiracy theories [79] and tried to redirect users searching for COVID-19–related information to official government sources [80].

YouTube facilitates the spread of misinformation to millions of viewers. It has been found that 27.5% of the most-viewed YouTube videos related to COVID-19 contained misinformation, and these reached over 62 million viewers worldwide [81]. About 17 million people subscribe to antivaccine accounts on YouTube [82]. As YouTube content is more difficult to create, it is probably less affected by bots sharing video content. However, bots can spread disinformation on YouTube through comments [83].

In the papers studied, the HPV vaccine was the second most common topic, after the topic of vaccines in general. This topic is popular in the discourse on antivaccine movements. The HPV vaccine has some specific features that make it vulnerable to theories that discourage vaccinations. First, in many countries, this vaccine is not mandatory; therefore, it can be more effectively discouraged. Second, the minimum age for receiving the first dose is 9 years; thus, often both parents’ consent and the preteen or teenager’s acceptance are required. Finally, apart from the ordinary antivaccination supporters, the HPV vaccine has other opponents. Conservative religious groups suggest that protection against sexually transmitted diseases encourages licentious teen sex. As a result, religious young women are undervaccinated and underinformed about the HPV vaccine [84].

### Future Research Propositions

Research conducted in languages other than English is limited. In our data set, only 2 articles analyzed non-English messages, which were in Italian [42,43]. We propose that a multilingual comparative study be undertaken to explore the similarities and differences in the vaccine-related discourse on social media between countries.

Another issue is the classification of messages as provaccine or antivaccine. Usually, this task is performed manually using codebooks. As this method is not scalable, we need to employ an automatic approach. Popularly, some of the messages are hand-labeled using a codebook, and machine learning models are applied to label the rest of the messages. We suggest that a universal codebook or dictionary be designed to assess whether a message has antivaccine sentiment. Such a tool would enhance the comparability of the research results.

Instagram is a social media platform that is still gaining popularity not only among users but also among scientists, as shown by the 2 articles from 2019 [39,49] that we included in our systematic review. However, previous Instagram studies have only examined opinions regarding HPV vaccines.

Analyzing data from private or secret Facebook groups [85] is important, as they often contain valuable information about users’ opinions and attitudes toward vaccinations. Although messages posted in such groups potentially contain higher quality information than publicly available data, they are not easy to obtain.

It should also be mentioned that the articles analyzed picture antivaccine messages related to specific words, phrases, and themes. Further research on these topics can result in the creation of effective tools to automatically detect fake news. This can help suppress vaccine hesitancy, which is connected to the spread of vaccine misinformation on social media [86].

### Limitations

First, we restricted the analysis to studies published between 2015 and 2019. As a result, some papers describing antivaccine messages on social media may not have been included. Second, we studied only articles written in English. Thus, we may have omitted articles published in other languages that analyzed non-English messages on social media.

### Conclusions

Antivaccine users create content that gains more positive reactions (likes, shares, retweets) on social media than provaccine messages do. Their messages are user friendly and well suited to the needs of users on social media platforms. Antivaccine users try to discourage vaccination using a few main arguments. They describe vaccines as harmful, highlight their side effects, and undermine the effectiveness of the protection they offer. To support these statements, they use false information and conspiracy theories, and gloss over or omit the data about vaccine benefits.

Public health authorities should continuously monitor social media to find new antivaccine arguments quickly and, based on that, design information campaigns targeting health professionals and ordinary users who are at a risk of being misinformed. Social media platforms have a big responsibility because they give millions of users access to misinformation. Knowledge of the characteristics of antivaccine content can help in the creation of tools that automatically tag false information. A positive trend in recent years is that social media platforms have attempted to stop the spread of vaccination misinformation.

## Abbreviations

HPV: human papillomavirus
PRISMA: Preferred Reporting Items for Systematic Reviews and Meta-Analyses
